# Sympathetic Nervous System Mediates Cardiac Remodeling After Myocardial Infarction in a Circadian Disruption Model

**DOI:** 10.3389/fcvm.2021.668387

**Published:** 2021-03-26

**Authors:** Yuhong Wang, Wanli Jiang, Hu Chen, Huixin Zhou, Zhihao Liu, Zihan Liu, Zhihao Liu, Yuyang Zhou, Xiaoya Zhou, Lilei Yu, Hong Jiang

**Affiliations:** ^1^Department of Cardiology, Renmin Hospital of Wuhan University, Wuhan, China; ^2^Cardiac Autonomic Nervous Research Center, Wuhan University, Wuhan, China; ^3^Department of Cardiology Cardiovascular Research Institute, Wuhan University, Wuhan, China; ^4^Hubei Key Laboratory of Cardiology, Wuhan, China; ^5^Department of Thoracic Surgery, Renmin Hospital of Wuhan University, Wuhan, China

**Keywords:** sympathetic nervous system, autonomic nervous system, cardiac dysfunction, circadian disruption, sympathetic ganglionic blockade

## Abstract

**Background:** Circadian rhythms have a considerable impact on the daily physiology of the heart, and their disruption causes pathology. Several studies have revealed that circadian disruption impaired cardiac remodeling after myocardial infarction (MI); however, the underlying brain-heart mechanisms remain unknown. We aim to discuss whether circadian disruption facilitates cardiac remodeling after MI by activating sympathetic nervous system.

**Methods:** Rats were randomly divided into three groups: Sham group (Sham), MI group (MI), and MI+ circadian disruption group (MI+Dis); rats were treated with pseudorabies virus (PRV) injections for trans-synaptic retrograde tracing; rats were randomly divided into two groups: MI+ circadian disruption + Empty Vector+ clozapine N-oxide (CNO) (Empty Vector), and MI+ circadian disruption + hM4D(Gi)+ CNO [hM4D(Gi)].

**Results:** Circadian disruption significantly facilitated cardiac remodeling after MI with lower systolic function, larger left ventricular volume, and aggravated cardiac fibrosis. Cardiac sympathetic remodeling makers and serum norepinephrine levels were also significantly increased by circadian disruption. PRV virus-labeled neurons were identified in the superior cervical ganglion (SCG), paraventricular nucleus (PVN), and suprachiasmatic nucleus (SCN) regions. Ganglionic blockade via designer receptors exclusively activated by designer drugs (DREADD) technique suppressed the activity of sympathetic nervous system and significantly alleviated the disruption-related cardiac dysfunction.

**Conclusion:** Circadian disruption adversely affected cardiac remodeling after MI possibly by activating sympathetic nervous system, and suppressing sympathetic activity can attenuate this disruption-related cardiac dysfunction.

## Introduction

Circadian rhythms are a physiological hallmark of the organism and are regulated by circadian clock networks that can synchronize body physiology and behavior to the environmental day-night cycle. An increasing number of studies have shown that the disruption of circadian rhythms confers a high risk for cardiovascular disease occurrence (1–4); cardiovascular disease can also cause circadian disruption, and this disruption could contribute to the occurrence of secondary cardiovascular events, including heart failure, acute myocardial infarction (MI), and stroke (5–7). It has been reported that aggravating the post-MI disruption by altering environmental light-dark cycle could worsen cardiac remodeling (8), whereas the beneficial effects of improving this disruption have also been shown (9, 10). Although circadian disruption plays a crucial role in the process of cardiac remodeling after MI, the underlying brain-heart mechanisms are still unclear.

The circadian clock networks have a master pacemaker in the suprachiasmatic nucleus (SCN), which can perceive environmental light through the retina and synchronize the oscillation of both its own clock and those in other organs and tissues to the environmental day-night cycle. One of the most important arms of the SCN in synchronizing the circadian clock networks is the sympathetic nervous system, and it is reported that light-induced SCN disorder could cause sympathetic activation (11). Our previous studies have demonstrated a strong connection between sympathetic activation and cardiovascular diseases (12–14). Increasing activity of the cardiac sympathetic nervous system adversely affects the ischemic myocardium, with increased infarct size, dilated atrium and ventricles, and impaired contractile and diastolic function (15, 16), while inhibiting cardiac sympathetic overactivity has been demonstrated to improve ventricular remodeling (12). Therefore, sympathetic activation may be an important link between circadian disruption and cardiac remodeling. Moreover, the superior cervical ganglion (SCG) is one of the most important sympathetic ganglia for the heart and previous studies have found that the descending projection from the paraventricular nucleus of the hypothalamus (PVN), a sympathetic integrative center that can receive projections from other regional nuclei and thus influence the sympathetic outflow to the periphery, to the SCG is involved in cardiovascular regulation (17, 18). These findings suggest a potential “SCN-PVN-SCG-Heart” sympathetic axis by which circadian rhythm information may be carried from the brain to the heart; however, anatomic or pathophysiologic evidence has not yet been uncovered.

In the search for neural mechanism by which the heart receives circadian information, we aim to identify the existence of an anatomic sympathetic axis between the SCN and the heart. And we also demonstrated the important role of this sympathetic axis in modulating cardiac remodeling after MI.

## Methods

### Animals

Experimental protocols were conducted under the guidelines of the National Institutes of Health and were approved by the Animal Care and Use Committees of Renmin Hospital of Wuhan University. Male Sprague-Dawley (SD) rats weighing 190–210 g were used in this work. Before MI model surgery, all rats were exposed to a regular 12:12 light-dark cycle for 14 days, and food and water were given *ad libitum*.

### Chronic MI Model

Rats were randomly divided into three groups (*n* = 8 each): (1) Sham group (Sham); (2) MI group (MI); (3) MI+ circadian disruption group (MI + Dis). The MI model was established by left anterior descending occlusion (LADO) surgery performed from 9 to 11 a.m. All rats were anesthetized with 2.5% sodium pentobarbital (50 mg/kg, i.p.) and were ventilated by a respirator after a tracheotomy. The left third intercostal space was opened to expose the heart, and then a pericardial incision was made to expose the left anterior descending coronary artery, which was ligated using 6–0 silk at 1–2 mm below the edge of the left auricle followed by closing the chest and skin using 4–0 silk. After surgery, MI rats recovered in a temperature-controlled cage at 37 degrees Celsius. Rats in the Sham group underwent the left thoracotomy without LADO.

### Circadian Disruption

Constant light (24 h/day) was used to induce circadian disruption, and rats were exposed to this abnormal light-dark cycle for only the first 7 days after MI model surgery. A 7-day constant light exposure was chosen for the present study based on the findings reported by Alibhai et al. (8) and Vazan (19), the former demonstrating the effects of a 5-day-light disruption on cardiac remodeling after MI and the latter demonstrating the adverse effects of constant light (24 h/day) on cardiac response to ischemia-reperfusion.

### Pseudorabies Virus (PRV) Trans-Synaptic Retrograde Tracer Technique

Rats were anesthetized with sodium pentobarbital and ventilated. The left third and fourth intercostal spaces were opened, and then a pericardial incision was made to expose the apex of the heart. PRV (PRV-CAG-EGFP, BrainVTA, Wuhan, China, 6^*^10^9^ pfu/ml, 0.5 μl/site) was injected directly into four sites of the ventricular myocardium in the apex region. These rats didn't receive the LADO. Following a 5-day survival period, rats were sacrificed and tissues including the heart, SCG, and brain were collected and fixed with paraformaldehyde followed by freezing slice process, which was used to determine the autofluorescence of PRV.

### Designer Receptors Exclusively Activated by Designer Drugs (DREADDs)

Rats were randomly divided into two groups: (1) MI+ Light disruption + Empty Vector+ CNO (Empty Vector, *n* = 5), and (2) MI+ Light disruption + hM4D(Gi)+ CNO [hM4D(Gi), *n* = 6]. Empty Vector (rAAV-hSyn-EGFP-WPRE-hGH pA, BrainVTA, Wuhan, China, 3.64^*^10^12^ vg/ml) and hM4D(Gi) virus [rAAV-hSyn-hM4D(Gi)-EGFP-WPRE-hGH pA, BrainVTA, Wuhan, China, 2.58^*^10^12^ vg/ml] were microinjected into the SCG in a volume of 1–2 μl. Rats were allowed to recover for 30 days before MI model surgery. A 7-day constant light exposure after MI model surgery was performed. Clozapine N-oxide (CNO) purchased from BrainVTA (Wuhan, China) was injected intraperitoneally at a dose of 3.3 mg/kg/d for 30 days after MI.

### Histological Staining

All rats were sacrificed 30 days after MI, and tissues including the heart, SCG, and brain were dissected quickly and then fixed with 4% paraformaldehyde in preparation for histological staining. To determine the ischemic injury, Masson staining (Servicebio, Wuhan, China) was performed in heart sections. Evaluating the expression of α-SMA (Servicebio, Wuhan, China) can reveal the degree of cardiac fibrosis. Tyrosine hydroxylase (TH, Abcam, Cambridge, Massachusetts) and synaptophysin (SYN, Life Technologies, Grand Island, New York) were used to assess the sympathetic activity (20). To identify the virus-EGFP positive cells in the SCG, TH expression was detected by immunohistochemical staining. The immunofluorescence of c-Fos (Servicebio, Wuhan, China) was used to evaluate the neuronal activity (21). The fluorescent dye DAPI (4′,6-diamidino-2-phenylindole) was used to locate the position of the nucleus. All analyses were quantitatively carried out with commercially available software (Image Pro Plus, Media Cybernetics, Inc., Rockville, MD).

### Determination of Serum Noradrenaline (NE) and Triglyceride (TG) Levels and Real-Time PCR

Blood samples from the aorta abdominalis were collected and stored at −80 degrees Celsius. The NE concentrations were determined with a Rat NE ELISA kit (CSB-E07022r, Cusabio Company, Wuhan, China), and the measurement procedure was carried out according to the manufacturer's instructions. Serum TG levels were determined using a Triglycerides GPO-PAP Kit (C061, Changchun Huili Biotech Co., Ltd., Jilin, China).

The mRNA expression levels of TGF-β1 in the heart were determined by real-time PCR. The following primer sequences were used: TGF-β1 (Forward Primer 5′-GGCGGTGCTCGCTTTGTA-3′, Reverse Primer 5′-TCCCGAATGTCTGACGTATTGA-3′) (22).

### Echocardiography and Monitoring of Blood Pressure and Heart Rate

Two-dimensional and M-mode echocardiography was conducted to assess the function and structure of the heart in all groups by using a High Resolution Imaging System (GE Vivid E95, USA) equipped with a 12-MHz probe (12S). Blood pressure and heart rate were monitored by noninvasive rat sphygmomanometer (BP-2010A, Softron Beijing Biotechnology Co., Ltd., Beijing, China).

### Measurement of Heart Rate Variability and Ventricular Fibrillation (VF) Threshold

Ten-minute electrocardiogram segments were recorded and analyzed by heart rate variability (HRV) module of LabChart computer software (ADInstruments, Castle Hill, NSW, Australia). Spectrum was integrated in very low (VLF: 0 to 0.2 Hz)-, low (LF: 0.20 to 0.75 Hz)- and high-frequency (HF: 0.75 to 2.5 Hz) bands. The high frequency (HF), the low frequency (LF), and the LF/HF ratio were calculated to evaluate cardiac sympathovagal balance. The VF-threshold was the minimal voltage that could induce VF. The pacing electrode was inserted in the left ventricular myocardium, and a train of pacing pulses (70 Hz, 1 ms) was delivered for 30 s. The pacing voltage of the first pulse started at 2.5 V and increments of 2.5 V steps were used until VF was induced.

### RNA Sequencing (RNA-Seq)

Detail description is available in the Supplementary Method.

### Statistical Analysis

Graphing and analysis were performed using GraphPad Prism software 7.0 (Inc., La Jolla, CA, USA). Data are shown as the means ± standard error of measurement (SEM) and the Shapiro-Wilk normality test was used to assess a normal distribution. Comparisons between groups were analyzed by one- or two-way ANOVA with the Bonferroni correction for the *post hoc* test, as well as the unpaired Student's *t*-test (two-tailed). Statistical significance was set at a *P*-value < 0.05.

## Results

### Circadian Disruption Facilitates Cardiac Function and Structure After MI

**Figure 1:**
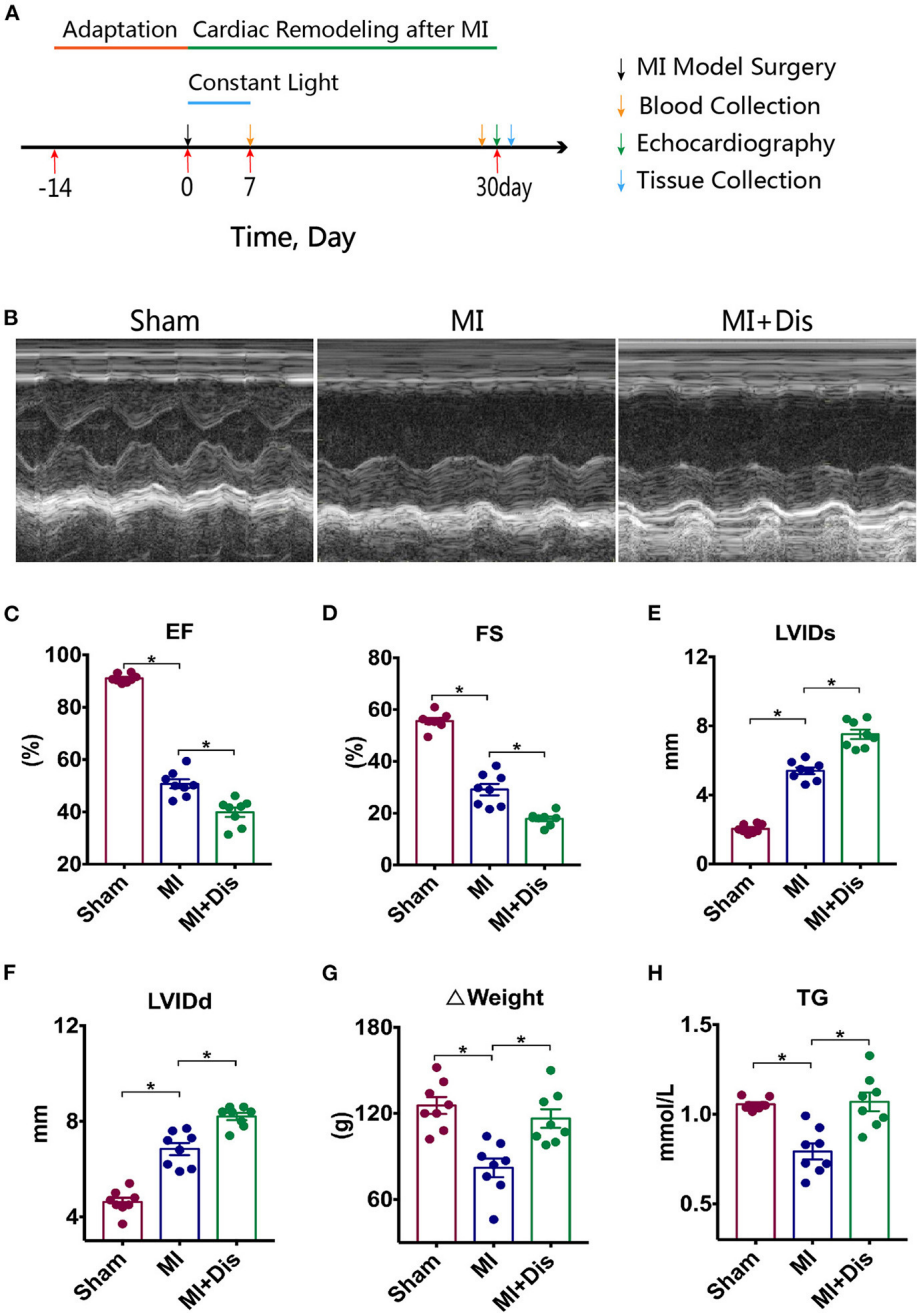
Cardiac function and structure were aggravated by circadian disruption after MI. Experimental protocol is outlined in **(A)**. Representative images of echocardiography in all groups are shown in **(B)**. **(C–F)** Differences of EF, FS, LVIDs, LVIDd among three groups. **(G)** Δweight (weight gain over the 30 days following MI). **(H)** Serum TG level. EF, ejection fraction; FS, fractional shortening; LVIDs, left ventricular internal dimension (systole); LVIDd, left ventricular internal dimension (diastole); TG, triglyceride; *p < 0.05.

We investigated whether altering environmental circadian rhythms caused different outcomes in cardiac function and structure which were assessed using echocardiography 30 days after MI. Experimental protocol was outlined in Figure 1A. Representative images of echocardiography in three groups were shown in Figure 1B. Ejection fraction (EF) and fractional shortening (FS) were usually used to evaluate the function of the heart, and both of these parameters were significantly decreased in the MI+Dis group compared with the MI only group (p^*^ < 0.05, Figures 1C,D). The left ventricular internal dimension (LVID) can indicate the volume of the left ventricle. MI rats with disruption showed larger systolic LVID (LVIDs) and diastolic LVID (LVIDd) than MI only rats (p^*^ < 0.05, Figures 1E,F). MI surgery caused an decrease in Δweight (weight gain over the 30 days following MI), while disruption was able to increase the weight gain compared with the MI only group (p^*^ < 0.05, Figure 1G). We further measured the serum TG, and results showed that MI rats with disruption showed higher level of TG than MI only rats (p^*^ < 0.05, Figure 1H).

### Circadian Disruption Facilitates Cardiac Fibrosis and Sympathetic Remodeling After MI

**Figure 2:**
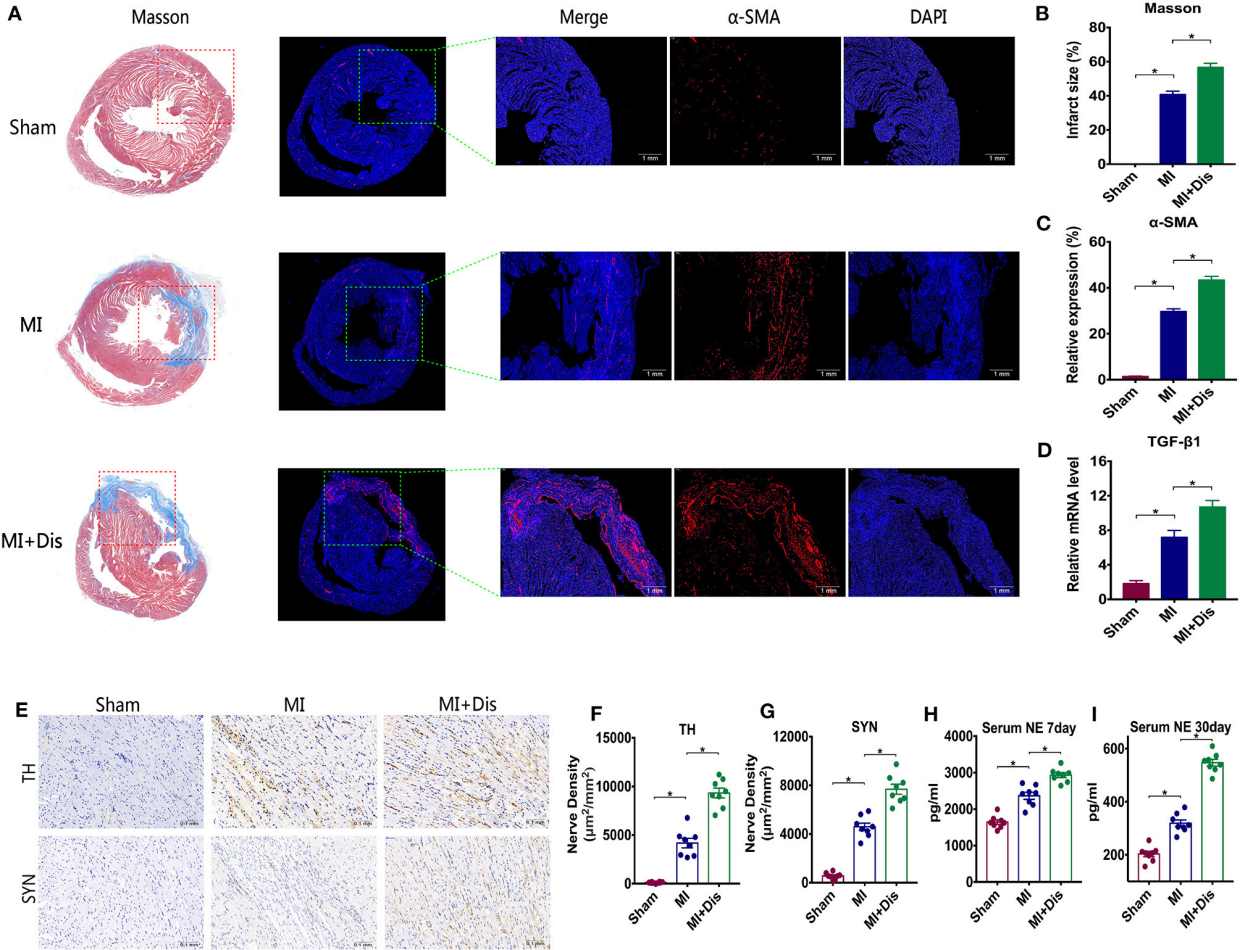
Cardiac fibrosis and sympathetic remodeling were aggravated by circadian disruption after MI. Representative images of staining for Masson (blue) and α-SMA (red) in all groups are shown in **(A)**, and quantitative analysis is shown in **(B,C)**. Real-time PCR results of TGF-β1 are shown in **(D)**. TH and SYN staining are used to assess sympathetic nerve sprouting and synaptic density in the heart **(E–G)**. **(H,I)** Serum NE levels. α-SMA, alpha smooth muscle actin; TGF-β1, transforming growth factor beta 1; NE, norepinephrine; TH, tyrosine hydroxylase; SYN, synaptophysin; p* < 0.05.

Compared with the MI only group, Masson and α-SMA staining detected a significant increase in positive expression in the heart after disruption (p^*^ < 0.05, Figure 2A) and quantitative evaluations were shown (p^*^ < 0.05, Figures 2B,C). Disruption also significantly facilitated the mRNA expression of TGF-β1 (p^*^ < 0.05, Figure 2D). We determined the changes of sympathetic nerves which richly innervates the heart. Immunohistochemical staining showed that circadian disruption led to higher cardiac sympathetic nerve sprouting and higher synaptic density, which were identified by TH and SYN markers (p^*^ < 0.05, Figures 2E–G). We used ELISA to detect the serum NE level at two time points: 7 and 30 days after MI. MI rats with disruption had a significant increase in serum NE levels both at 7 and 30 days after MI (p^*^ < 0.05, Figures 2H,I).

### Circadian Disruption Facilitates Cardiac Remodeling at the Transcriptional Level

We used RNA sequencing to determine the most significant changes induced by circadian disruption at the myocardial transcriptional level. The results of principal component analysis (PCA) and expression level heat-map identified that the cardiac transcriptome of rats with MI only was different from that in rats with MI plus circadian disruption (Supplementary Figures 1A,B). It was found that disruption could dramatically change the genes of “Environmental Information Processing” and its “Signal transduction” (Supplementary Figure 1C). And then, we analyzed the results of GO functional enrichment and the top 8 GO terms were displayed (Q-value < 0.05, Supplementary Figure 1D). Disruption could significantly change the extracellular matrix, cardiac muscle contraction, lipid metabolism, and inflammation of the heart (Supplementary Figure 1D). Importantly, environmental circadian disruption significantly altered the cardiac genes belonging to cardiac clock genes (Arntl, Npas2, Ciart, Dbp), cardiac nervous system (Robo2, Brinp2, Psd, Bmp5, Ntrk3, and Gdf10), cardiac muscle contraction (Actn3 and Atp2a1), extracellular matrix (Ltbp2, Thbs4, Wisp2, Nov, Comp), inflammation (Mif, Defb1, Spp1, Hamp) and lipid metabolism (Angptl4, Mrap, Angptl8, C1qtnf3) (p^*^ < 0.05, p^**^ < 0.01, Supplementary Figures 1E–J).

### The Heart Receives Multi-Synaptic Neural Input From the SCN

**Figure 3:**
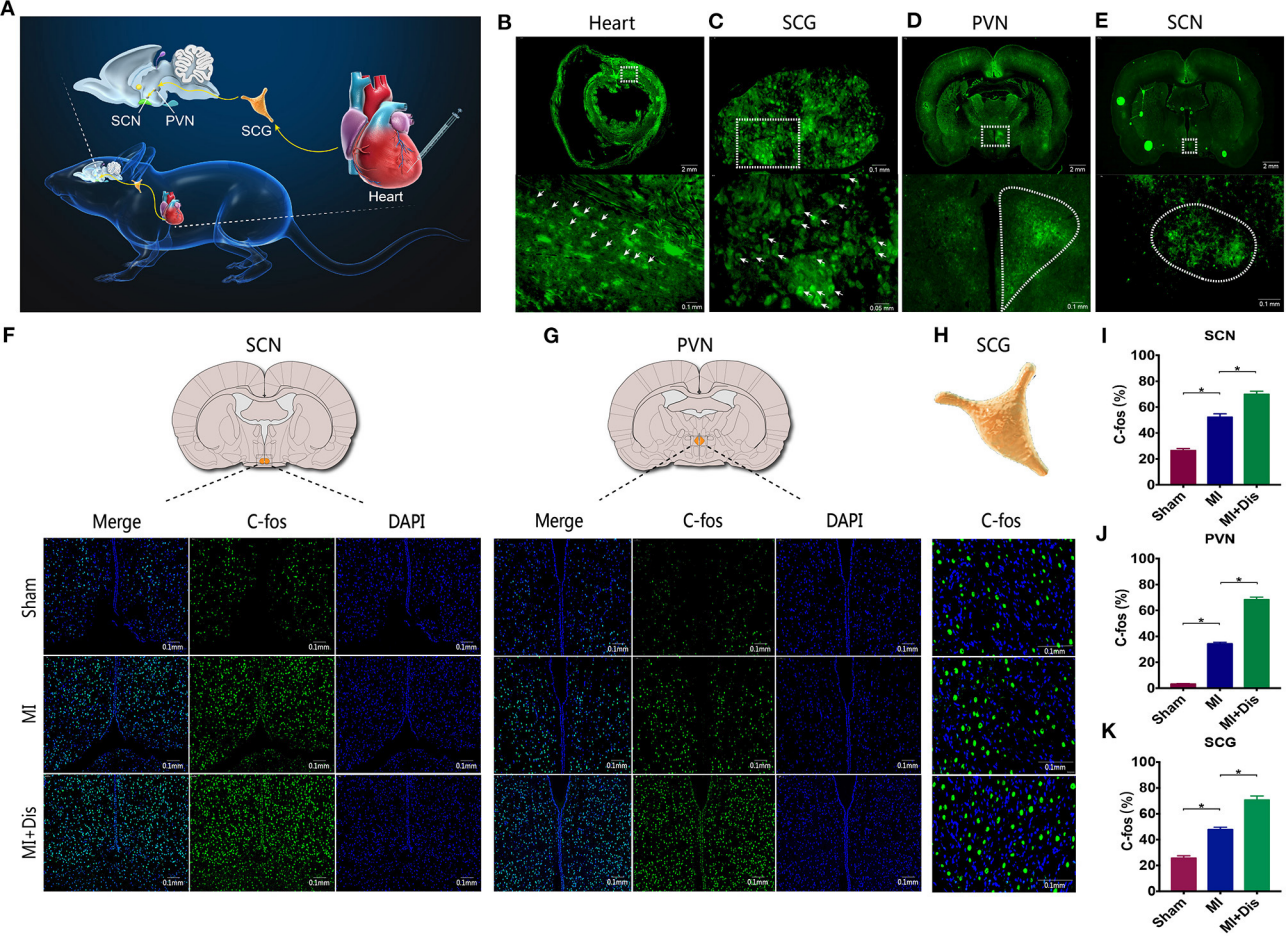
Circadian disruption activated the “SCN-PVN-SCG-Heart” sympathetic axis. Retrograde tracer virus is injected into the ventricular myocardium in the apex region **(A)**, and labeled neurons were identified in the heart **(B)**, SCG **(C)**, PVN **(D)**, and SCN **(E)** regions. **(F–K)** Expression levels of c-fos at the SCN, PVN, and SCG sites. EGFP, enhanced green fluorescent protein; SCN, suprachiasmatic nucleus; PVN, paraventricular nucleus of hypothalamus; SCG, superior cervical ganglion; p* < 0.05.

To demonstrate the presence of neural connections between circadian master pacemaker SCN and the heart, we injected a trans-synaptic retrograde tracer virus PRV into the ventricular myocardium in the apex region (Figure 3A). Cardiac injection of PRV resulted in the appearance of PRV-labeled neurons in the myocardium (Figure 3B). Moreover, labeled neurons were also found in the SCG, PVN, and SCN region after cardiac injection of PRV (Figures 3C–E). These findings supported the presence of an anatomic connection between circadian master pacemaker SCN and the heart, that is, the “SCN-PVN-SCG-Heart” sympathetic axis. Furthermore, we found circadian disruption could induce a significant increase in expression levels of c-Fos at all three regions (p^*^ < 0.05, Figures 3F–H) and quantitative evaluations were shown (p^*^ < 0.05, Figures 3I–K), indicating that circadian disruption was able to activate this sympathetic axis.

### Ganglionic Blockade by DREADD

**Figure 4:**
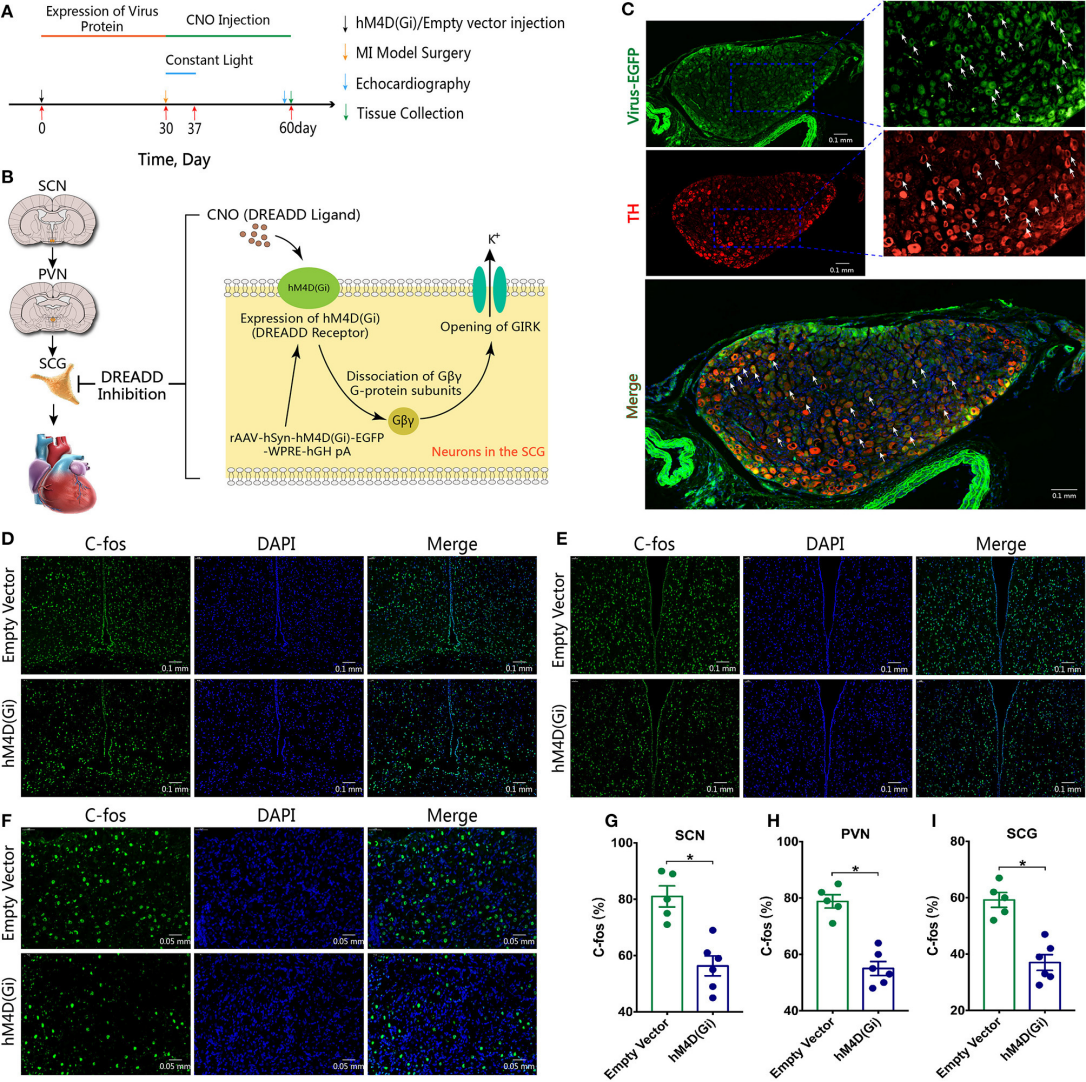
Sympathetic ganglionic blockade by DREADD. **(A)** Experimental protocol of DREADD. **(B)** Rationale of DREADD. **(C)** virus-EGFP (green) and TH (red). **(D–I)** Expression levels of c-Fos at the SCN, PVN, and SCG. CNO, Clozapine N-oxide; GIRKs, G-protein sensitive inwardly rectifying potassium channels; p* < 0.05.

Experimental protocol and rationale of DREADD were outlined (Figures 4A,B). DREADD technology is widely used in neuroscience and it is an important tool for the modulation of neural functions. Neurons expressing hM4D(Gi) in the SCG can be activated by the synthetic ligand CNO exposure, and thus leading to dissociation of Gβγ G-protein subunits and opening of G-protein sensitive inwardly rectifying potassium channels (GIRKs) (Figure 4B) (23). Double immunofluorescence staining for virus-EGFP (green) and TH (red) verified that hM4D(Gi) was successfully expressed in the sympathetic neurons located in the SCG (Figure 4C). Expression levels of c-Fos at the SCN, PVN, SCG were significantly decreased in the hM4D(Gi) group (p^*^ < 0.05, Figures 4D–I).

### Effects of Ganglionic Blockade on Cardiac Dysfunction in a Circadian Disruption Model

**Figure 5:**
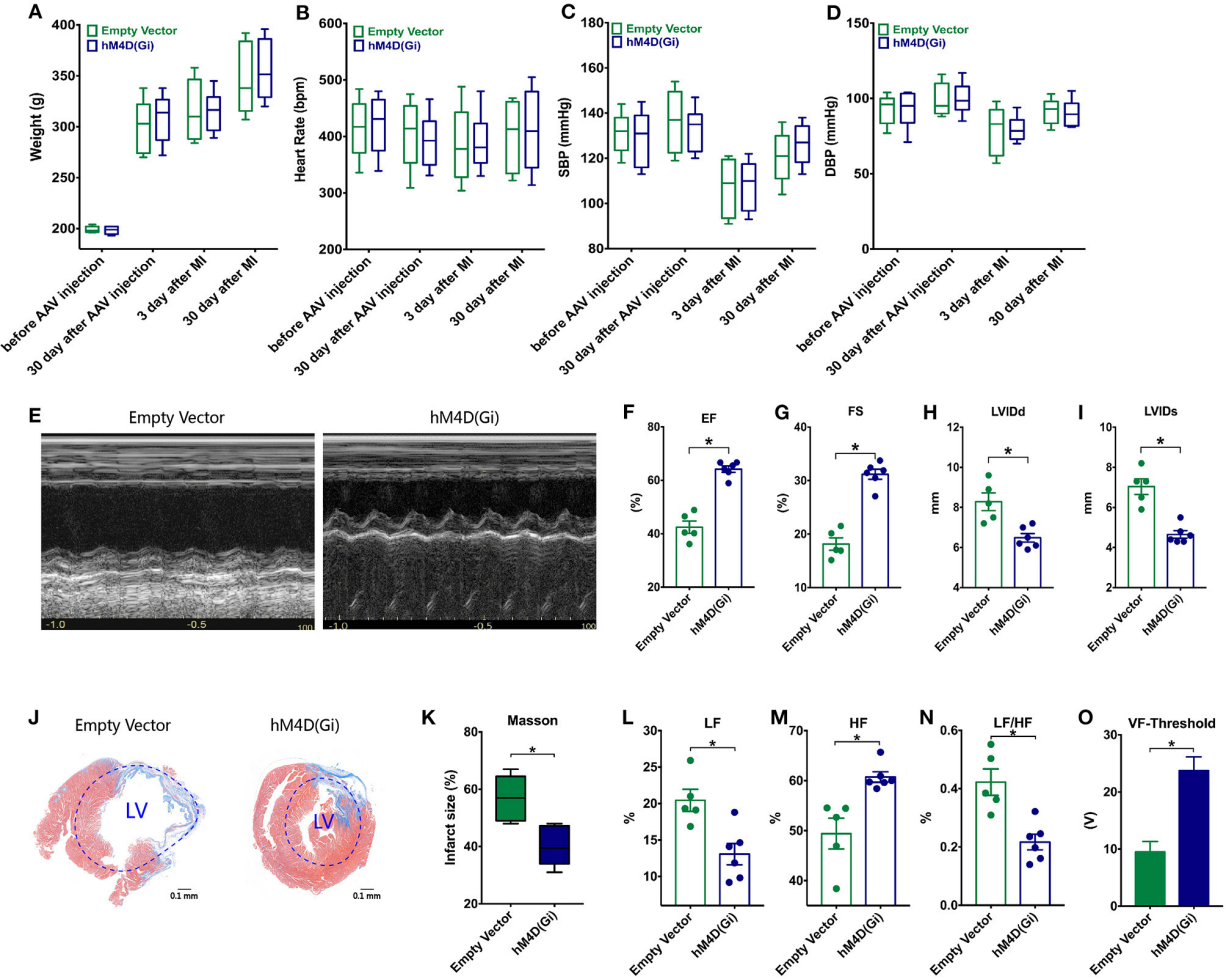
Effects of ganglionic blockade on cardiac dysfunction in a circadian disruption model. **(A–D)** Difference between weight, heart rate, systolic blood pressure (SBP) and diastolic blood pressure (DBP) of two groups. **(E)** Representative images of echocardiography. **(F–I)** Differences of EF, FS, LVIDs, and LVIDd. **(J,K)** Masson staining. **(L–N)** HRV: LF, HF and LF/HF. **(O)** VF-Threshold. HRV, Heart rate variability; LF, low frequency; HF, the high frequency, the LF/HF ratio, and ventricular fibrillation, VF; p* < 0.05.

No significant difference between weight, heart rate, systolic blood pressure (SBP) and diastolic blood pressure (DBP) of two groups was observed at different time points (*p* > 0.05, Figures 5A–D). Representative images of echocardiography in two groups were displayed (Figure 5E). MI rats with ganglionic blockade showed higher EF, higher FS, and smaller LVID than rats with control virus (p^*^ < 0.05, Figures 5F–I). Ganglionic blockade also improved cardiac fibrosis, which was evaluated by Masson staining (p^*^ < 0.05, Figures 5J,K). Moreover, ganglionic blockade was able to significantly decrease the LF and the LF/HF ratio and markedly enhance the HF, indicating that cardiac sympathetic activity was suppressed (p^*^ < 0.05, Figures 5L–N). Rats with ganglionic blockade had a higher Ventricular Fibrillation (VF)-Threshold compared with the Empty Vector group, suggesting that the instability of ventricular electrophysiology was reduced by ganglionic blockade (p^*^ < 0.05, Figure 5O).

We used RNA sequencing to determine the most significant changes induced by cardiac sympathetic ganglionic blockade at the myocardial transcriptional level. The results of PCA and heat-map expression identified the differences of cardiac transcriptomes between two groups (Supplementary Figures 2A,B), and gene number of each sample was showed (Supplementary Figure 2C). Compared with the Empty Vector group, 76 genes were up-regulated while 52 genes were down-regulated in the hM4D(Gi) group (Supplementary Figure 2D). Among this genes, ganglionic blockade can significantly improve the disorder of cardiac clock genes (Per2, Per3, Arntl, Cry2, Npas2, Ciart, Dbp), inhibit cardiac nervous system remodeling (Robo2, Syngap1, Neo1, NGF, Ntrk3), and attenuate cardiac remodeling (Myh6, Tpm2, Cox5b, Ctgf, Eln, Fbn1) (p^*^ < 0.05, p^**^ < 0.01, p^***^ < 0.001, Supplementary Figures 2E–G).

## Discussion

**Figure 6:**
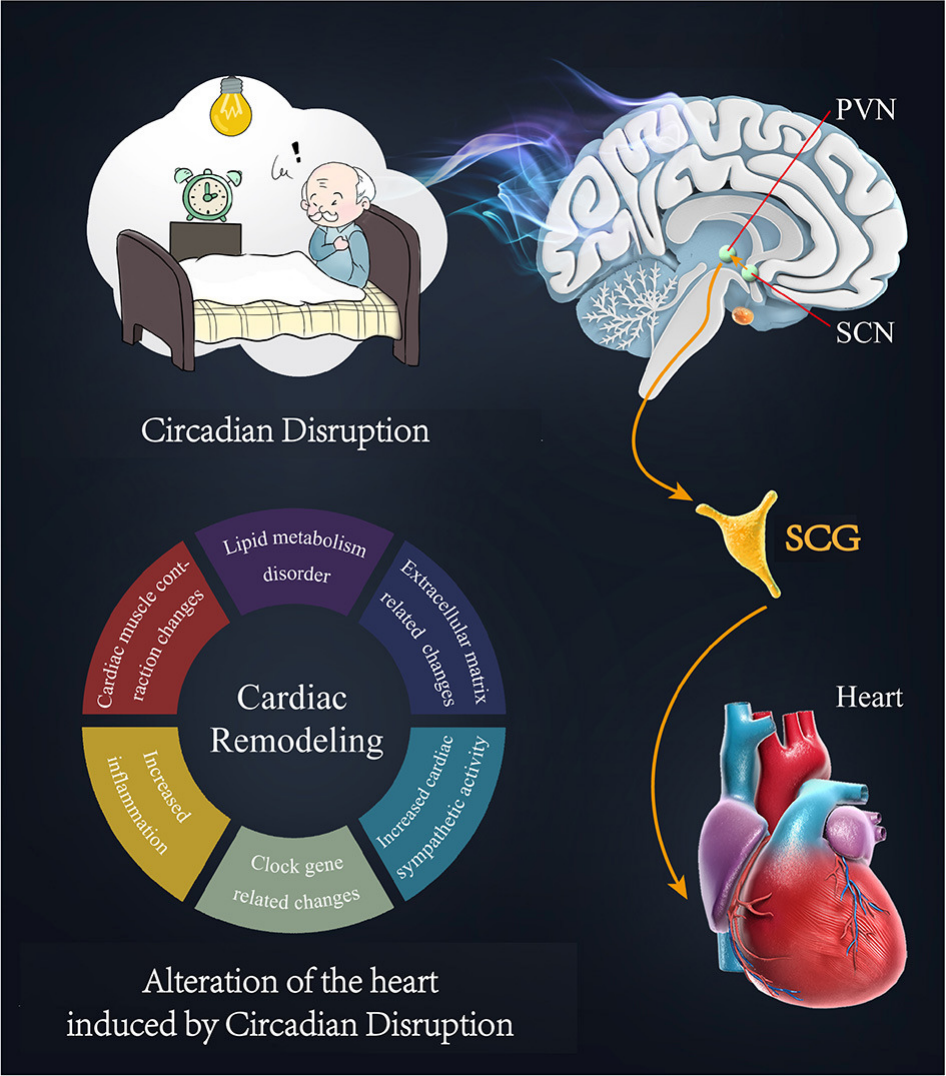
Sympathetic nervous system is the key link between circadian disruption and cardiac remodeling. Circadian disruption aggravates cardiac remodeling after MI possibly by activating sympathetic nervous system. Circadian information may be carried to the heart by the “SCN-PVN-SCG-Heart” sympathetic axis mechanism. Cardiac remodeling at the transcriptional level is aggravated by circadian disruption, and the changes mainly include cardiac local clock, cardiac nervous system, cardiac muscle contraction, extracellular matrix, lipid metabolism, and inflammation.

In this study, we found that circadian disruption adversely affected cardiac repair after MI possibly by activating sympathetic nervous system. To provide a direct anatomic evidence, we used a neuro-tracing technique to determine the existence of a sympathetic axis between circadian master pacemaker SCN and the heart. And results showed that circadian disruption was capable of activating this sympathetic axis, while suppressing the axis could attenuate the circadian disruption-related cardiac dysfunction. Thus, sympathetic nervous system is essential for transmitting circadian information from central clock to the heart. Modulating sympathetic activity may become a novel potential neuromodulation strategy to treat the circadian disruption-related cardiac dysfunction (Figure 6).

Growing evidence has shown that circadian disruption affects cardiovascular physiology with an increase in heart rate and heart rate variability, suggesting an important role of circadian disruption in cardiovascular health (24). Disruption induced by altering light-dark cycle can significantly affect cardiac local clock rhythm and contributed to progression of cardiac dysfunction (25). A preclinical study showed that disruption was able to aggravate the cardiac structure and function after MI and adversely affect long-term outcome (8). Consistently, in this study, we also found that circadian disruption was an indeed negative factor for cardiac remodeling after MI. For a more comprehensive understanding of disruption effects on the heart, we further used the RNA-Seq to determine the significant changes in the transcriptome of the heart, and found that disruption had profound influences on cardiac local nervous system and clock genes, as well as lipid metabolism and inflammation.

Many cardiac local mechanisms of disruption are uncovered, but there should be an upstream mechanism by which the heart receives center circadian information. The SCN is the master pacemaker of circadian rhythm, and one of its most important arms in synchronizing the circadian clock is the sympathetic nervous system (11). Evidence has shown that light can cause disorder of the SCN and induce an increase of arterial blood pressure, heart rate, and renal sympathetic nerve activity, indicating that a light-disturbed output of the SCN affects the sympathetic control of peripheral organ function (11). It has also been demonstrated that the SCN controls the circadian rhythm of cardiac function via sympathetic nervous system (26, 27). Moreover, heart is richly innervated by sympathetic nervous system and our previous studies have identified the adverse role of sympathetic activation in the progression of cardiac dysfunction (12–14). These findings suggest a sympathetic connection between circadian disruption and cardiac dysfunction. In this study, we identified the crucial role of sympathetic activation in circadian disruption-related cardiac remodeling, and retrograde tracer results revealed a “SCN-PVN-SCG-Heart” sympathetic axis possibly by which the heart received environmental circadian information.

Cardiac sympathetic ganglion is a key component of sympathetic nervous system and the direct recording of ganglion nerve activity reveals that its sympathetic tone exhibits circadian rhythmicity (28–32). It is known that cardiac autonomic nervous system is divided into the extrinsic and intrinsic nervous system, and extrinsic cardiac sympathetic system includes preganglionic and postganglionic neurons as well as their axons (33). Preganglionic neurons are located within the brain and postganglionic neurons outside the brain includes the SCG (C1–3), stellate ganglia (C7-T2) and thoracic ganglia (T2-7), which can mediate the pathophysiology of cardiac performance and thus being known as cardiac sympathetic ganglions (34). Our previous studies have demonstrated the key role of cardiac sympathetic ganglion in the initiation and maintenance of cardiovascular diseases, and found that suppressing the activation of cardiac sympathetic ganglion was capable of reducing the incidence of adverse cardiac events (14, 35–37). And in this study, we found that suppressing cardiac sympathetic ganglion was capable of improving circadian disruption-related cardiac remodeling, resetting cardiac sympathovagal balance, and reducing the instability of ventricular electrophysiology; and sympathetic ganglionic blockade can also significantly improve the disorders of cardiac local clocks at the transcriptional level. All these results suggested that suppressing cardiac sympathetic ganglion, a key target of cardiac sympathetic outflow, can attenuate the adverse effects of circadian disruption on cardiac remodeling.

Circadian disruption is capable of affecting lipid metabolism. Lipidomics results have verified the circadian regulation of lipid metabolism (38), and feeding-related nuclei in the hypothalamus have been identified to receive input from the circadian master pacemaker SCN (39) and hence provide a direct mechanism for circadian regulation of lipid metabolism. Furthermore, circadian disruption was able to increase food intake and weight gain in mice and could significantly increase the serum levels of triglyceride, cholesterol, leptin, and glucose (40). Several studies have shown that mice with deletion of Bmal1 exhibit lipid metabolism disorders, with increased body fat content and hyperlipidemia (41). Similar conclusions were shown in this study, and we found lipid metabolism related genes, including Angptl4, Mrap, Angptl8, and C1qtnf3, were significantly changed by disruption, suggesting that disruption induced lipid metabolism disorders in the myocardium. Furthermore, our results also revealed that disruption was capable of increasing weight gain and inducing hypertriglyceridemia.

### Study Limitations

As a preliminary study to investigate the neural connection between circadian disruption and cardiac remodeling, we only demonstrated the key role of the “SCN-PVN-SCG-Heart” sympathetic mechanism in chronic MI model. More information about its actions in other cardiovascular disease models, like I/R injury, hypertension, and arrhythmia, may provide a better understanding of this brain-heart sympathetic axis. Moreover, we sequenced the cardiac transcriptome for a comprehensive understanding of the effects of circadian disruption on the ischemic heart, which may well provide a starting framework to further investigate more exact mechanisms of circadian disruption-related cardiac dysfunction.

### Clinical Implication

Sympathetic nervous system is essential for transmitting circadian information from central clock SCN to the periphery, and sympathetic neuromodulation strategy may become a novel strategy for cardiac circadian disruption.

## Data Availability Statement

The original contributions presented in the study are included in the article/Supplementary Material, further inquiries can be directed to the corresponding author/s.

## Ethics Statement

The animal study was reviewed and approved by Animal Care and Use Committees of Renmin Hospital of Wuhan University.

## Author Contributions

HJ, LY, and YW designed the study, analyzed data, and wrote the manuscript. YW, HC, and HZ collected laboratory data. WJ and ZhL (fifth author) performed the statistical analysis. XZ, ZiL, ZhL (seventh author), and YZ edited manuscript. All authors contributed to the article and approved the submitted version.

## Conflict of Interest

The authors declare that the research was conducted in the absence of any commercial or financial relationships that could be construed as a potential conflict of interest.

## Abbreviations

MI: Myocardial infarction
PRV: Pseudorabies virus
CNO: Clozapine N-oxide
SCG: Superior cervical ganglion
PVN: Paraventricular nucleus
SCN: Suprachiasmatic nucleus
DREADD: Designer receptors exclusively activated by designer drugs
LADO: Left anterior descending occlusion
TH: Tyrosine hydroxylase
SYN: Synaptophysin
NE: Noradrenaline
TG: Triglyceride
VF: Ventricular fibrillation
HRV: Heart rate variability
HF: High frequency
LF: Low frequency
EF: Ejection fraction
FS: Fractional shortening
LVIDs: Left ventricular internal dimension (systolic)
LVIDd: Left ventricular internal dimension (diastolic)
PCA: Principal component analysis
SBP: Systolic blood pressure
DBP: Diastolic blood pressure.
