# Supplementary material for: Sympathetic Nervous System Mediates Cardiac Remodeling After Myocardial Infarction in a Circadian Disruption Model
Source: Front Cardiovasc Med. 2021 Mar 26;8:668387. doi: 10.3389/fcvm.2021.668387 (PMC8032890; doi:10.3389/fcvm.2021.668387)

Supplemental Methods

RNA sequencing (RNA-Seq)

The ischemic myocardium of rats was quickly dissected and snap-frozen in liquid nitrogen for storage. The sample quality was assessed using an Agilent 2100 Bioanalyzer with a kit (Agilent RNA 6000 Nano Reagents Part 1), and samples with 28S/18S less than 1.0 or RQN (RNA quality number) less than 7.0 were discarded. Total RNA was enriched using magnetic beads with OligodT, and then a single-stranded circular DNA library was prepared for the following RNA-Seq. Briefly, the sequencing reads were mapped to the rat genome (Rattus_norvegicus, UCSC, rn6) using HISAT (Hierarchical Indexing for Spliced Alignment of Transcripts), and reads were also mapped to rat genes using Bowtie2 (version 2.2.5; -q --phred64 --sensitive –dpad 0 --gbar 99999999 --mp 1,1 --np 1 --score-min L,0,-0.1 -p 16 -k 200). To identify the gene expression levels of samples, Bowtie2 was used for mapping, and RSEM (version1.2.8; default) was used to calculate the expression level. To identify the differential gene expression between groups, the DEGseq2 algorithm was used with the predefined parameters "Fold Change ≥ 2 and Adjusted P-value (Q-value) ≤ 0.001". The Pheatmap function from the R software package was applied to depict these differentially expressed genes as a heatmap. Gene ontology (GO) enrichment and Kyoto Encyclopedia of Genes and Genomes (KEGG) pathway enrichment were analyzed using the phyper function from the R software package, and the P-value algorithm was given as follows:

$$P = 1 - \sum_{i=0}^{m-1} \frac{\binom{M}{i}\binom{N-M}{n-i}}{\binom{N}{n}}$$

The P-value was adjusted by FDR, and the adjusted P-value was designated as the Q-value. The significance level was set at an FDR ≤ 0.01. More details can be found in supplemental files.

Supplemental Figures

Supplementary Figure S1. Cardiac remodeling at the transcriptional level was aggravated by circadian disruption. Q-values < 0.05. PCA, Principal component analysis; KEGG, Kyoto Encyclopedia of Genes and Genome; GO, Gene Ontology; Q-value, FDR-adjusted P-value; FPKM, fragments per kilobase of transcript per million fragments mapped; $p^{*}<0.05$, $p^{**}<0.01$.

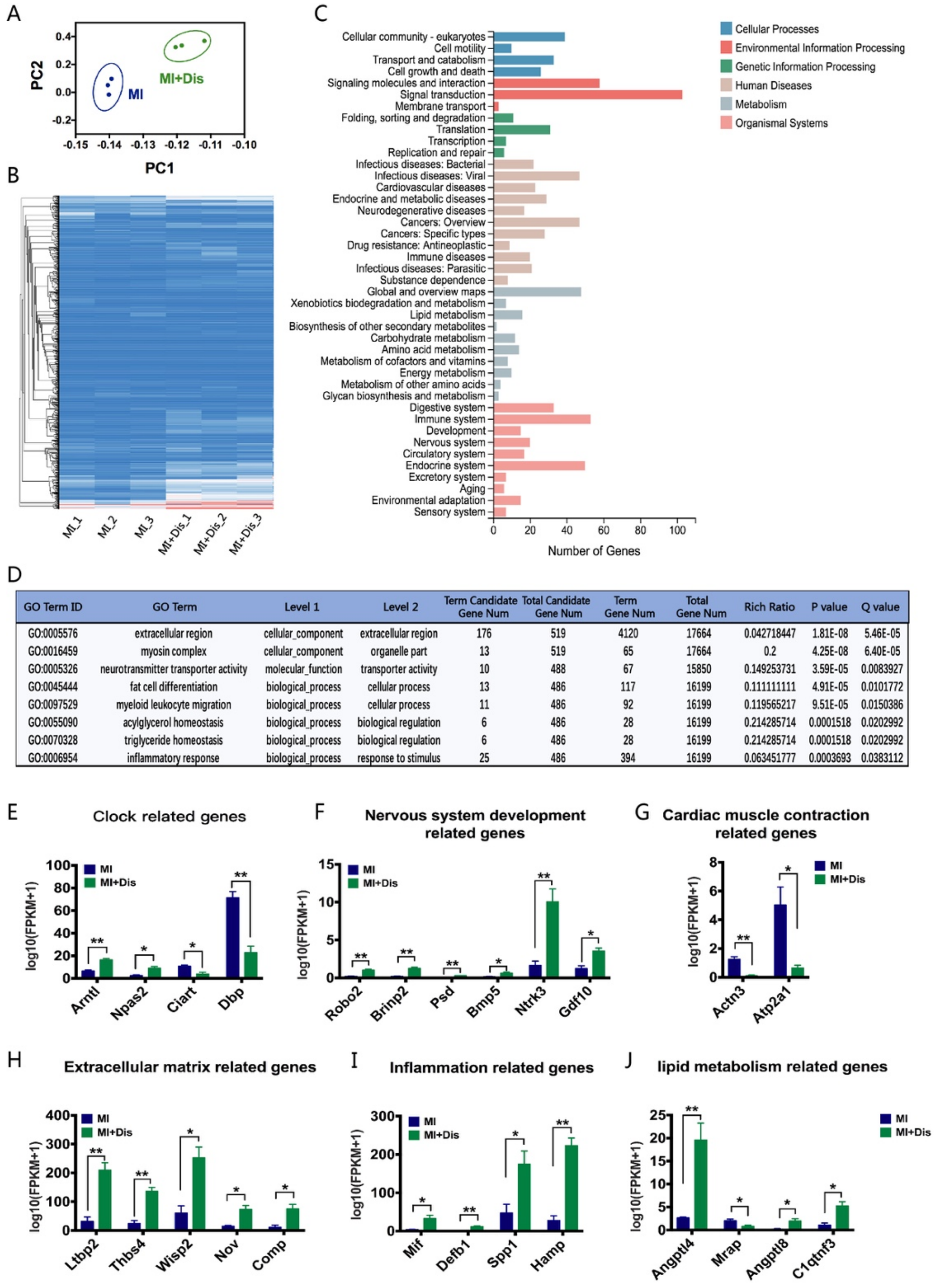

GO Term ID	GO Term	Level 1	Level 2	Term Candidate Gene Num	Total Candidate Gene Num	Term Gene Num	Total Gene Num	Rich Ratio	P value	Q value
GO:0005576	extracellular region	cellular_component	extracellular region	176	519	4120	17664	0.042718447	1.81E-08	5.46E-05
GO:0016459	myosin complex	cellular_component	organelle part	13	519	65	17664	0.2	4.25E-08	6.40E-05
GO:0005326	neurotransmitter transporter activity	molecular_function	transporter activity	10	488	67	15850	0.149253731	3.59E-05	0.0083927
GO:0045444	fat cell differentiation	biological_process	cellular process	13	486	117	16199	0.111111111	4.91E-05	0.0101772
GO:0097529	myeloid leukocyte migration	biological_process	cellular process	11	486	92	16199	0.119565217	9.51E-05	0.0150386
GO:0055090	acylglycerol homeostasis	biological_process	biological regulation	6	486	28	16199	0.214285714	0.0001518	0.0202992
GO:0070328	triglyceride homeostasis	biological_process	biological regulation	6	486	28	16199	0.214285714	0.0001518	0.0202992
GO:0006954	inflammatory response	biological_process	response to stimulus	25	486	394	16199	0.063451777	0.0003693	0.0383112

Supplementary Figure S2. Effects of ganglionic blockade on cardiac remodeling at the transcriptional level. $p^{*}<0.05$, $p^{**}<0.01$, $p^{***}<0.001$.

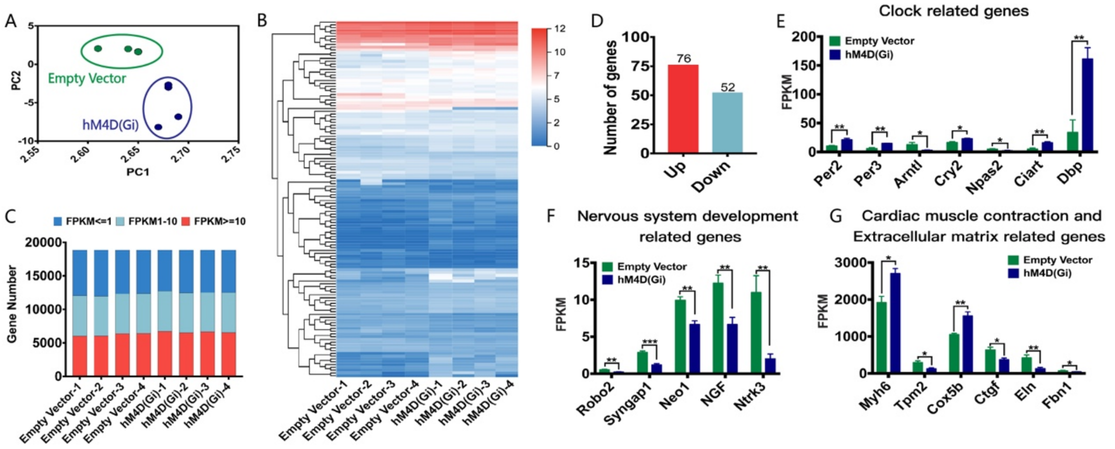

Supplement: Supplementary file 1 [file Data_Sheet_1.PDF]
